# Computer-assisted catalyst development via automated modelling of conformationally complex molecules: application to diphosphinoamine ligands

**DOI:** 10.1038/s41598-021-82816-x

**Published:** 2021-02-25

**Authors:** Sibo Lin, Jenna C. Fromer, Yagnaseni Ghosh, Brian Hanna, Mohamed Elanany, Wei Xu

**Affiliations:** 1 Aramco Americas - Boston Research Center, 400 Technology Square, Cambridge, MA 02139 USA; 2 Department of Chemical and Biological Engineering, Tufts University, Medford, MA 02155 USA; 3 Chemicals R&D Division, Research and Development Center, Saudi Aramco, Dhahran, 31311 Saudi Arabia; 4 Chemicals R&D Lab at KAUST, Research and Development Center, Saudi Aramco, Thuwal, 23955 Saudi Arabia

**Keywords:** Homogeneous catalysis, Computational chemistry, Density functional theory, Structure prediction

## Abstract

Simulation of conformationally complicated molecules requires multiple levels of theory to obtain accurate thermodynamics, requiring significant researcher time to implement. We automate this workflow using all open-source code (XTBDFT) and apply it toward a practical challenge: diphosphinoamine (PNP) ligands used for ethylene tetramerization catalysis may isomerize (with deleterious effects) to iminobisphosphines (PPNs), and a computational method to evaluate PNP ligand candidates would save significant experimental effort. We use XTBDFT to calculate the thermodynamic stability of a wide range of conformationally complex PNP ligands against isomeriation to PPN (ΔG_PPN_), and establish a strong correlation between ΔG_PPN_ and catalyst performance. Finally, we apply our method to screen novel PNP candidates, saving significant time by ruling out candidates with non-trivial synthetic routes and poor expected catalytic performance.

## Introduction

**Figure 1:**
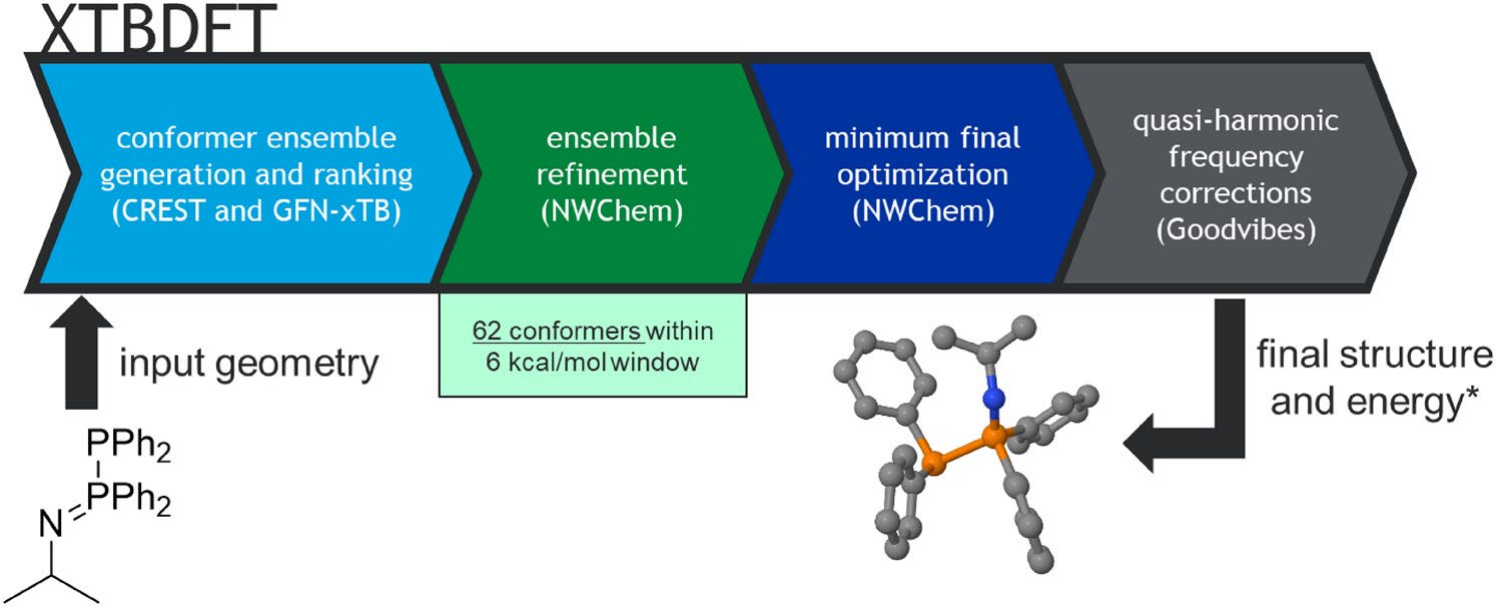
XTBDFT flowchart with example input and output.

Quantum mechanical methods with high energy accuracy, such as density functional theory (DFT), can optimize molecular input structures to a nearby *local* minimum, but calculating accurate reaction thermodynamics requires finding *global* minimum energy structures^1,2^. For simple molecules, expert intuition can identify a few minima to focus study on, but an alternative approach must be considered for more complex molecules or to eventually fulfil the dream of autonomous catalyst design^3,4^: the potential energy surface must be first surveyed with a computationally efficient method; then minima from this survey must be refined using slower, more accurate methods; finally, for molecules possessing low-frequency vibrational modes, those modes need to be treated appropriately to obtain accurate thermodynamic energies^5–7^. This multistep process has a prohibitively steep learning curve for many newcomers, and trained researchers spend significant time monitoring calculations and transferring data from one phase to the next. Various programs have been previously written to automate the workflow between computational chemistry engines^8–15^. We have constructed our own Python script, XTBDFT, to automate the workflow between (1) GFN-xTB-driven^16^ CREST^2,12,17^, an accurate and efficient meta-dynamics method for conformational analysis for systems, particularly those with transition-metal atoms and exotic functional groups, (2) conformer refinement with DFT, as implemented in NWChem^18^, and (3) GoodVibes, a script to apply quasi-harmonic treatment of low-frequency vibrational modes^6^ (Fig. 1). Notably all components of this automated workflow are open-source, allowing for widespread and affordable implementation. Herein we apply XTBDFT toward a practical topic: computational assessment of diphosphinoamine (PNP) ligand candidates.

**Figure 2:**
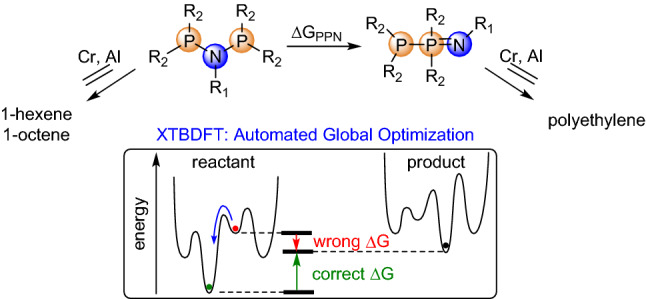
Diphosphinoamine (PNP) to iminobisphosphine (PPN) isomerization and illustrative potential energy surfaces.

PNP ligands are extensively studied for Cr-catalyzed ethylene oligomerisation to valuable linear alpha olefins^19–22^. Recent academic^23–25^ and industrial^26^ studies continue to show the prominence of PNP ligands. However, reactor fouling, caused by insoluble polyethylene by-product, remains a major impediment to industrial-scale practice. PNP ligands can isomerize to iminobisphosphines (PPN) under catalytic conditions, which has been proposed to lead to increased polyethylene formation^24^. We sought to evaluate that hypothesis by calculating thermodynamic stabilities of PNPs against isomerization to PPN form (ΔG_PPN_, Fig. 2). Both PNP and PPN molecules possess many conformations, and analysis of the wrong conformer will result in incorrect ΔG_PPN_. A previous DFT study of PNP ligand conformers found variation of up to 9.9 kcal/mol in calculated Gibbs free energy^25^, which would drastically impact any thermodynamic comparison with PPN isomer.

**Figure 3:**
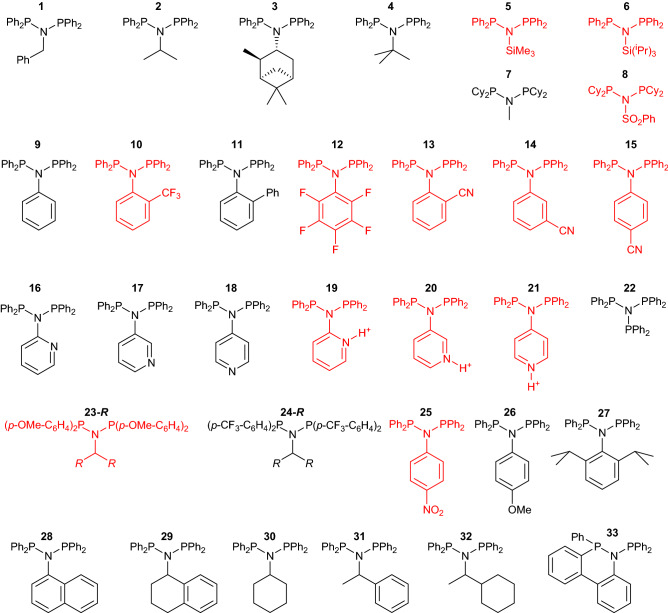
PNP ligands **1**–**33** or PPN isomers (**1′**–**33′**, not shown) that have been reported in the literature. Black structures are calculated to have positive ΔG_PPN_; red structures have negative ΔG_PPN_.

In this report, we utilize XTBDFT to automate global optimization and calculation of ΔG_PPN_ of several known PNP/PPN compounds (**1–33**, Fig. 3), and observe strong agreement with experimental observations. Then a strong inverse relation between PNP stability and polyethylene formation during ethylene oligomerization catalysis is observed. Finally, this method is applied to screen novel PNP ligand candidates, saving significant time by ruling out candidates with non-trivial synthetic routes and poor expected catalytic performance.

## Methods

Initial guess geometries for PNP and PPN molecules were conveniently generated with MolView^27^. These molecules have numerous hindered degrees of rotation, and standard geometry optimization algorithms may yield a relatively high-energy conformer. A previous DFT study of relatively simple (i.e. high substitutional symmetry) PNP molecules found that geometric local minima could differ by 9.9 kcal/mol in Gibbs free energy^25^. Human-directed generation of conformer ensembles for computational analysis is time-intensive and inconsistent. To ensure identification of a low-energy conformer, an efficient computational method must be utilized to generate and crudely rank a broad conformer ensemble. Conformer sampling algorithms employed in Tinker^28^, OpenBabel^29^, and various other molecular modelling programs rely on heavily parameterized classical force fields or semiempirical methods that may not accurately reproduce geometries or energies of exotic molecules or transition-metal complexes, and in some cases researchers have to resort to substituting unparameterized elements with more common elements^30^. We instead use the meta-dynamics package Conformer Rotamer Ensemble Sampling Tool (CREST^2,17^) driven by the semi-empirical density functional tight binding theory GFN2-xTB, which, unlike previously developed semi-empirical methods, has been broadly parameterized for all elements H-Rd and benchmarked against diverse chemical databases^16^, to automate generation of an ensemble of conformers within 2.0 kcal/mol of the minimum energy conformer. As an added benefit of using CREST for conformation searching, its built-in conformer symmetry analysis identifies rotamers that are chemically identical, greatly reducing the size of the conformer ensemble to be processed by higher levels of theory. In one example, CREST identified 35 unique conformers of (Ph_2_P)_2_NPh (**9**) within 6 kcal/mol of the lowest energy conformer. For comparison, the conformer searching procedure built into Spartan ‘18 (powered by Merck Molecular Force Field^31^) returns 225 conformers.

Further geometry optimizations of the conformer ensemble were performed with density functional theory (DFT) as implemented in NWChem^18,32^ (version 6.8) with the B3LYP functional^33,34^, def2-SV(P) basis set^35^, Weigend Coulomb-fitting auxiliary basis set^36^, Grimme DFT-D3 dispersion corrections^37^, NWChem medium integration grid, and loose geometric convergence criteria (using NWChem input parameters of gmax 0.002; grms 0.0003; xrms 1; xmax 1). The lowest energy conformer at this intermediate level of theory was then further geometry optimized with tighter convergence criteria (gmax 0.0001; grms 0.00003; xrms 0.0006; xmax 0.001), and thermochemical corrections were calculated, with the def2-SVP basis set^35^ and the NWChem fine integration grid. Finally, a high-level single-point electronic energy evaluation was performed using the def2-TZVP basis set^35^.

Because of the hindered degrees of rotation, several optimized geometries possess low-magnitude frequency vibrations, which are inaccurately treated by the harmonic oscillator approximation for thermodynamics. A correction for these vibrations was applied by quasi-harmonically raising all frequencies below 100 cm^−1^ using the GoodVibes script^6^. Goodvibes code was modified to allow parsing of NWChem output files, and this customization has been merged into the publicly available Goodvibes release.

Taking advantage of the programmable nature of CREST, NWChem, and GoodVibes input files, a Python wrapper script was written to automate all the steps starting from an initial guess geometry (.xyz file). This script (XTBDFT) communicates data between the various programs, tracks calculations, and automatically triggers the next calculation in the procedure. While code to interface CREST and GFN-xTB with DFT programs such as Orca and Turbomole has been previously published^12,13^, those programs are not as freely licensed and distributed as NWChem. NWChem is open-source and freely licensable for all users, which has facilitated its implementation in massively distributed cloud-computing solutions^38–40^. While molecular dynamics has been interfaced with NWChem^11^, the underlying molecular mechanics forcefields are not accurately parameterized for transition metal atoms or exotic functional groups. We believe the portable and lightweight nature of this wrapper script, along with the generous licensing terms of the underlying free chemistry engines, will allow for wide-spread adoption and modification among the chemistry and molecular machine-learning communities. The current version of XTBDFT is available online^41^.

## Results

### Composite procedure for identifying lowest-energy conformer

To computationally predict the thermodynamics of PNP to PPN isomerization, the minimum-energy conformation of each isomer must be obtained. Conventional geometry optimization algorithms employed by DFT software may identify a relatively high-lying local minimum, especially in systems containing multiple hindered degrees of rotation. Thus, we developed a composite procedure to identify and evaluate a low-energy conformation, consisting of: (a) the semi-empirical quantum chemical meta-dynamics package CREST to generate and crudely rank a diverse ensemble of conformers and (b) a quick, low level of DFT to re-optimize and re-rank the lowest energy conformers. The composite procedure is flexible in the choices of thermochemical recipe for determining the lowest-energy conformation.

**Figure 4:**
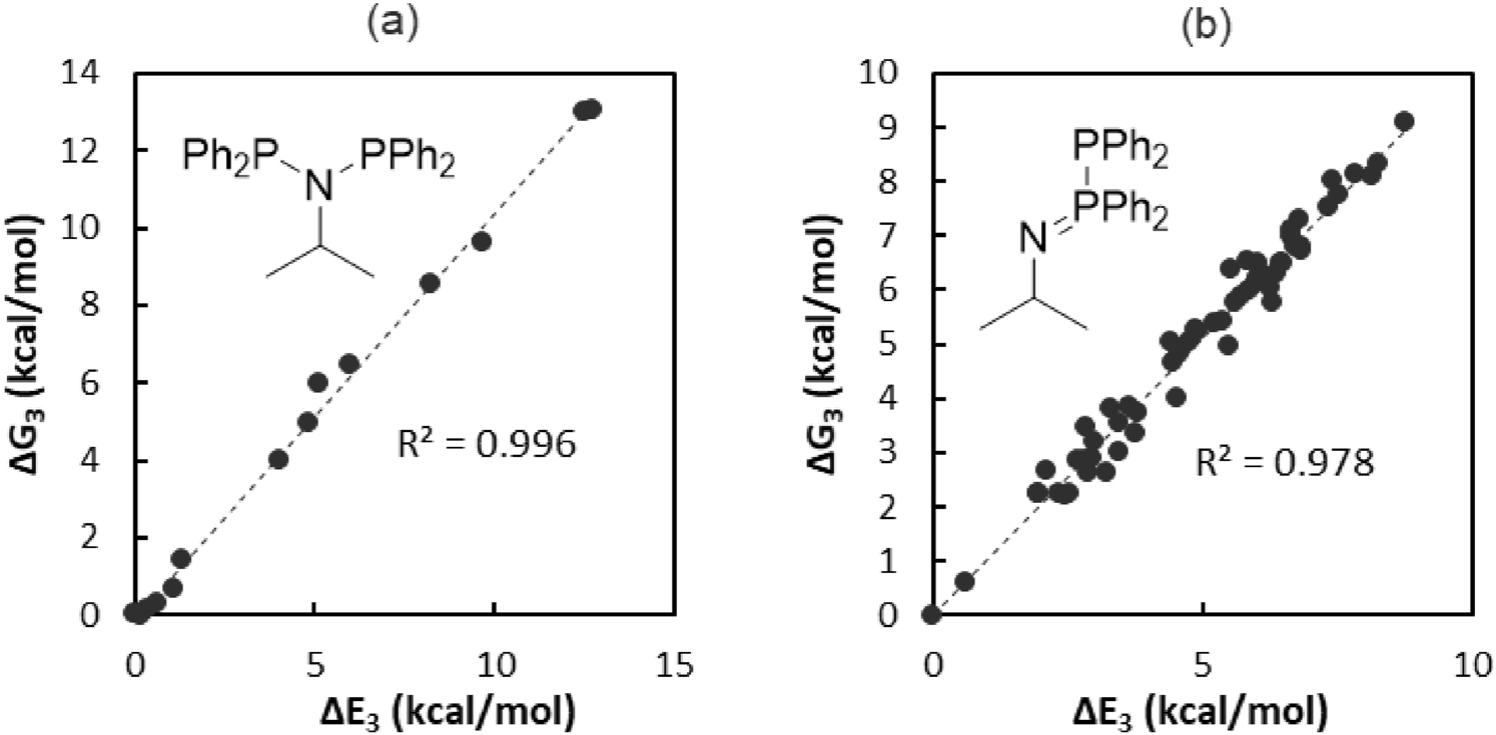
Comparison of relative free energies of conformers (ΔG_3_) versus relative electronic energies of conformers (ΔE_3_) for (**a**) **2** and (**b**) **2′.**

As a prototypical case, we considered the conformer ensembles (ΔE_XTB_ < 6 kcal/mol vs. minimum-energy conformer) of (Ph_2_P)_2_N^i^Pr (**2**) and its PPN isomer **2′**. Free energy corrections, G_xtb_(RRHO), were calculated using GFN2-xTB vibrational calculations on the GFN2-xTB-optimized structures. A variety of electronic energies were obtained for each conformer, E_0-3_ (Table 1), of increasing computational cost. For analysis, we consider relative energies:

1 $$\Delta {\text{E}}_{n} = {\text{ E}}_{n} {-}{\text{ E}}_{n}^{0} ,$$

where E_*n*_^0^ is the electronic energy of the minimum conformer in the initial CREST search. ΔE_3_ is strongly correlated with ΔG_3_ (Fig. 4) for conformer ensembles of both **2** (R^2^ = 0.996) and **2′** (R^2^ = 0.978); thus vibrational calculations were not employed in identifying the lowest-energy conformers for other compounds in this study.

**Table 1:** Thermochemical recipes.

	Energy calculation	Geometry optimization
E_xtb_	E(GFN2-xTB)	GFN2-xTB
E_0_	E(B3LYP-D3/def2-SV(P))	GFN2-xTB
E_1_	E(B3LYP-D3/def2-SV(P)	B3LYP-D3/def2-SV(P)^a^
E_2_	E(B3LYP-D3/def2-SVP)	B3LYP-D3/def2-SV(P)^a^
E_3_	E(B3LYP-D3/def2-SVP)	B3LYP-D3/def2-SVP^a^
G_0-3_	E_0-3_ + G_xtb_(RRHO)	
E_4_	E(B3LYP-D3/def2-TZVP)	B3LYP-D3/def2-SVP^b^
G_4_	E_4_ + G_qh_(B3LYP-D3/def2-SVP)	

^a^Loose NWChem geometry convergence criteria: gmax 0.002; grms 0.0003; xrms 1; xmax 1.

^b^Tight NWChem geometry convergence criteria: gmax 0.0001; grms 0.00003; xrms 0.0006; xmax 0.001.

**Figure 5:**
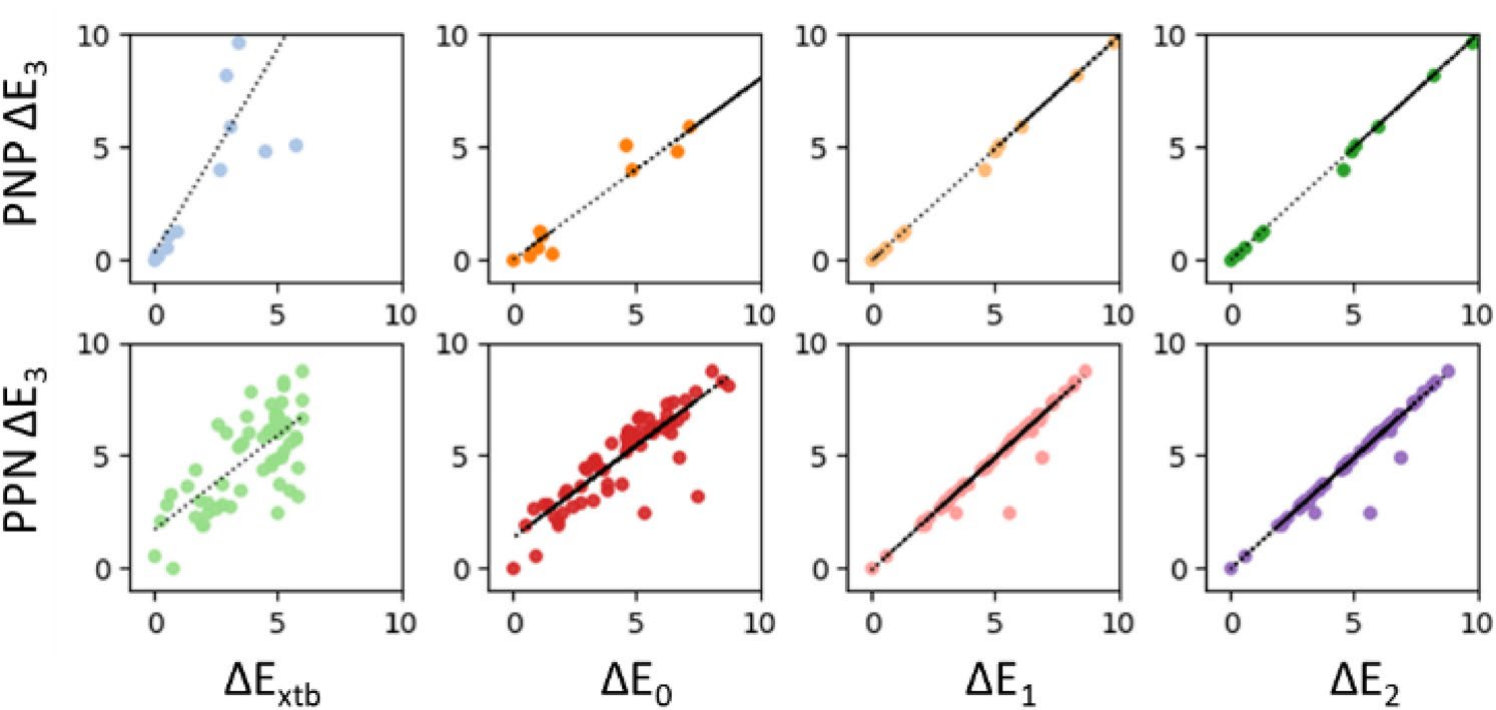
Comparison of thermochemical recipes against ΔE_3_ for conformer ensembles of **2** and **2′** (all units are kcal/mol).

Further computational savings could be achieved by using a lower level of theory for geometry optimization and electronic energy evaluation. For the conformer ensembles of **2** and **2′**, we plotted the ΔE_3_ of each conformer versus its ΔE_XTB_ and ΔE_0-2_ (Fig. 5). The energy calculations ΔE_1_ and ΔE_2_ were highly correlated with ΔE_3_ (R^2^ > 0.94), indicating that the computational expense of expanding the basis set from def2-SV(P) to def2-SVP for single-point energy evaluations is not required. In contrast, the more affordable energy calculation ΔE_0_ was accurate for the conformer ensemble of **2** (R^2^ = 0.98), but not for that of **2′** (R^2^ = 0.75). The most affordable energy calculation, ΔE_xtb_, was inaccurate for ordering conformer ensembles of both **2** and **2′**. GFN-xTB was developed to produce accurate *g*eometries, *f*requencies, and *n*on-covalent interactions with remarkable computational efficiency^16^, but for accurate relative energy calculations in this study, higher-level DFT calculations are necessary to determine global minimum energy conformations. Thus, ΔE_1_ was chosen as an economical yet accurate computational metric by which to identify the lowest energy conformer.

### PNP-to-PPN isomerization energy (ΔG_PPN_) of known compounds

The above procedure identified the lowest energy conformer for a wide range of PNP compounds and their PPN isomers. The lowest energy conformer was further optimized with B3LYP-D3/def2-SVP, and electronically evaluated using B3LYP-D3/def2-TZVP (E_4_). Quasi-harmonic thermochemical corrections were applied to obtain free energies for the lowest energy PNP and PPN conformers, G_4_(PNP) and G_4_(PPN). The free energy of PNP-to-PPN isomerization was then calculated:

2 $$\Delta {\text{G}}_{{{\text{PPN}}}} = {\text{ G}}_{4} \left( {{\text{PPN}}} \right) \, {-}{\text{ G}}_{4} \left( {{\text{PNP}}} \right),$$

The ΔG_PPN_ of **1**–**33** are tabulated in Table 2 and found to match experimental observations (see “Discussion” section).

**Table 2:** Calculated PNP-to-PPN isomerization energy (ΔG_PPN_ , kcal/mol).

PNP	ΔG_PPN_	PNP	ΔG_PPN_	PNP	ΔG_PPN_
**1**	8.6	**16**	0.6	**31**	6.7
**2**	9.5	**17**	2.5	**32**	7.5
**3**	9.5	**18**	1.4	**33**	6.1
**4**	2.5	**19**	− 13.2	**34**	0.4
**5**	− 2.6	**20**	− 9.3	**35**	− 6.3
**6**	− 10.4	**21**	− 12.2	**36**	0.6
**7**	12.5	**22**	0.6	**37**	0.3
**8**	− 1.7	**23 –CH**_**3**_	− 0.1	**38**	0.7
**9**	3.5	**24 –CH**_**3**_	11.6	**39**	0.3
**10**	− 4.3	**25**	− 0.9	**40**	− 6.0
**11**	2.5	**26**	4.7	**41**	− 3.2
**12**	− 1.0	**27**	3.8	**42**	0.1
**13**	− 2.8	**28**	0.5	**43**	− 3.9
**14**	− 0.4	**29**	8.9	**44**	− 7.8
**15**	− 0.1	**30**	9.0		

### Screening novel compounds

**Figure 6:**
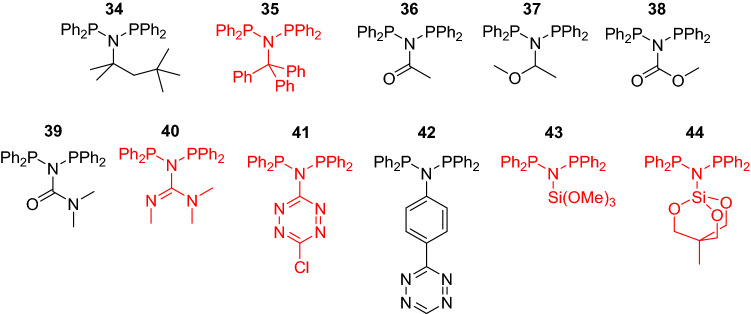
Novel ligand candidates with ΔG_PPN_ < 1 kcal/mol. Black structures are calculated to have positive ΔG_PPN_; red structures have negative ΔG_PPN_.

Having benchmarked our composite computational method against reported experimental observations, we applied this method to predict the synthetic accessibility of novel PNP ligand candidates. Screening a wide pool of candidates, several were predicted to be thermodynamically stable against isomerization to PPN (ΔG_PPN_ > 1 kcal/mol); synthesis and catalytic testing of these candidates is underway and will be reported in a future publication. Some examples of ligand candidates with ΔG_PPN_ < 1 kcal/mol (Table 1) are shown in Fig. 6 (**34**–**44**) and listed in Table 2.

## Discussion

To assess the accuracy of our computational method, we compared ΔG_PPN_ for **1**–**33** to previously reported experimental observations. PNP/PPN isomers can be kinetically trapped during synthesis from lithiated aminophosphine and chlorophosphine; however, in some cases the kinetic products can be observed converting to the thermodynamic product in the presence of excess chlorophosphine, which acts as an isomerization catalyst^42,43^. Alternatively, synthesis from primary amine, two equivalents of chlorophosphine, and triethylamine in dichloromethane solvent is proposed to yield the thermodynamic product because PNP-PPN isomerization is catalyzed by triethylammonium chloride^42^. We thus expected the calculated ΔG_PPN_ values (Table 1) to correspond with previously reported experimental observations.

The PNPs derived from alkylamines (**1**–**4**) have been isolated via the triethylamine route^22,42,44^, and accordingly have positive calculated ΔG_PPN_. *N*-trimethylsilylamine-derived PNP **5** has only been synthesized with the lithiation route^45,46^, even in studies where other PNP compounds were made via the triethylamine route^46^; this observation is consistent with the calculated ΔG_PPN_ of − 2.6 kcal/mol. With bulker *N*-triisopropylsilylamine, **6** has never been isolated. Using the same synthetic procedure as for **5**, only the PPN isomer **6′** was observed, which is consistent with the much more negative ΔG_PPN_ of − 10.4 kcal/mol. With bis(dicyclohexylphosphino)amines, the ΔG_PPN_ values match previously isolated isomers **7** and **8′**^43^. For aniline-based molecules, ΔG_PPN_ agrees with experimentally observed isomers from triethylamine syntheses: **9**, **10′**, **11**, **12′**^47^, and **13′**^47^. Depending on the synthetic conditions, different PNP/PPN product mixtures have been reported for **14** and **15**^47^, and accordingly these compounds have relatively small but negative ΔG_PPN_. The *N*-pyridyl compounds **16**–**18** were all isolated from triethylamine syntheses, and they all exhibit positive ΔG_PPN_. Their reported isomerization to PPN species upon protonation^48^ is matched by the negative ΔG_PPN_ for **19**–**21**. Tris(diphenylphosphino)amine **22** has been synthesized in only 8% yield from lithiated bis(diphenylphosphino)amine and chlorophosphine^49^, although other reports have reported exclusive formation of the PPNP isomer **22′**^45^. ΔG_PPN_ is small (0.6 kcal/mol), reflecting the accessibility of both isomers.

**23-(n-C**_**17**_**H**_**35**_**)** and **24-(n-C**_**17**_**H**_**35**_**)** have both been isolated from triethylamine-based syntheses^25^. While ΔG_PPN_ of **23-CH**_**3**_ has a small negative value, that of **24-CH**_**3**_ is quite positive and large, leading to the novel observation: *electron-withdrawing P-substituents favour the PNP isomer*. We expect this to be a useful design strategy for novel PNP ligands.

Conversely, *N*-substitution follows the opposite pattern, as shown by NO_2_-substituted **25** and OMe-substituted **26** with ΔG_PPN_ of − 0.9 and 4.7 kcal/mol, respectively. The negative ΔG_PPN_ of **23-CH**_**3**_ and **25** conflicts with the reportedly isolated PNP compounds, and perhaps indicate that the error of this computational thermodynamic method is *ca*. 1 kcal/mol. Compounds **27**–**32** have all been isolated in the PNP form from triethylamine syntheses, and accordingly they all have ΔG_PPN_ > 0. These similarly sized compounds clearly show that ΔG_PPN_ is higher with *N*-alkyl substitution instead of *N*-aryl substitution.

As a strategy to disfavour PPN isomerization, the N-substituent can be covalently tethered to a *P*-substituent. Synthesis of **33** was reported to yield no detectable amounts of **33′**^50^, and accordingly **33** has a ΔG_PPN_ of 6.1 kcal/mol (*c.f.* 3.5 kcal/mol for **9**).

Summarizing the results for our computational method, with |ΔG_PPN_|> 0.9 the predicted PNP/PPN isomer matches experimentally reported isomers synthesized with triethylamine. With |ΔG_PPN_|≤ 0.9 kcal/mol, the experimentally isolated isomers depend on exact experimental conditions. Compare this accuracy with conventional DFT geometry optimization to a single nearest minimum: for PNP molecules, conformers 9.9 kcal/mol higher than the minimum energy conformer have been identified previously^25^.

Having established the agreement of ΔG_PPN_ with experimental observations, we sought to examine its relation to polyethylene by-product formation during Cr-catalyzed ethylene oligomerization^24^. These catalytic reactions are known to be highly sensitive to air, moisture, temperature, and various experimental parameters, and so we selected experimental data previously published by Sasol that were all collected under similar conditions^44,46,51^. There is a notable correlation between ΔG_PPN_ and lower polyethylene formation (Table 3). As a simplified model, if PNP and PPN isomers are in thermodynamic equilibrium ([PPN]_0_/[PNP]_0_ = e^−ΔGPPN/RT^), initial PNP concentration is directly correlated with ethylene oligomerization productivity (oligomerization productivity = *a*[PNP]_0_), initial PPN concentration is directly correlated with polymerization productivity (polymerization productivity = *b*[PPN]_0_), and *a* and *b* are constant across the range of PNP and PPN compounds, then the following relation should be observed:

3 $$\ln \, \left( {{\text{polyethylene}}\,{\text{wt}}\% } \right) \, = \, \ln \, \left( {b{/}a} \right) \, {-} \, \Delta {\text{G}}_{{{\text{PPN}}}} {\text{/ RT}}$$

**Figure 7:**
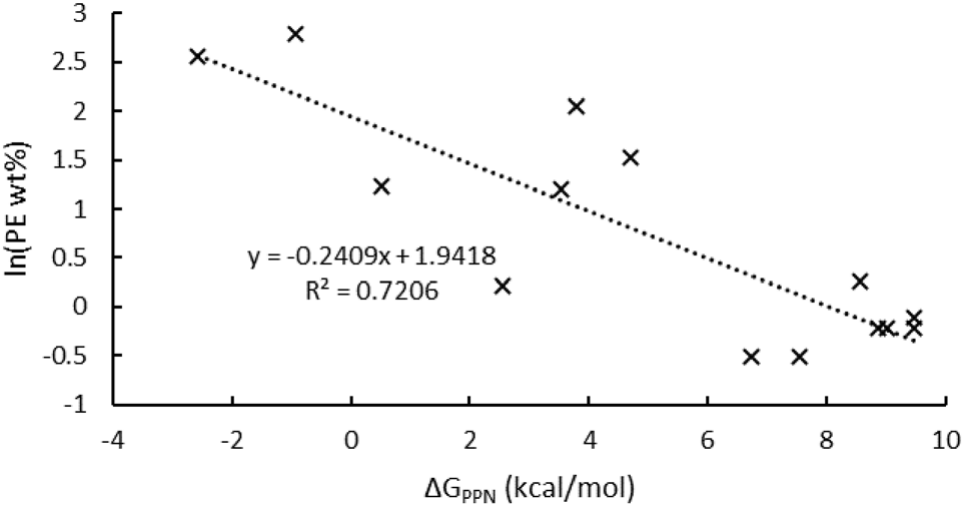
Reported polyethylene by-product production versus ΔG_PPN_ in graphical form.

In agreement, there is an inverse linear relationship between the logarithm of polyethylene wt% and ΔG_PPN_ (Fig. 7), supporting the proposal that polyethylene formation proceeds through PPN-derived catalytic species. Scatter results from *a* and *b* varying across the range of ligands (experimental catalytic activities, listed in Table 3, span two orders of magnitude between the various ligands). However, there is still remarkable correlation using this simplified model (R^2^ = 0.72), indicating that PNP stability against isomerization to PPN is a useful design criterion for novel ligands.

**Table 3:** Reported polyethylene production (wt.%) versus ΔG_PPN_ (kcal/mol).

ΔG_PPN_ (kcal/mol)	Polyethylene wt%	Ligand	Activity (kg/(g_Cr_h))
− 2.6	12.9	**5**	20.1
− 0.9	16.2	**25**	932.8
0.5	3.4	**28**	516.9
2.5	1.2	**4**	1550
3.5	3.3	**9**	765.9
3.8	7.8	**27**	159.6
4.7	4.6	**26**	385.9
6.7	0.6	**31**	1001.6
7.5	0.6	**32**	3200
8.6	1.3	**1**	1070
8.9	0.8	**29**	145.5
9.0	0.8	**30**	2150
9.5	0.9	**2**	1950
9.5	0.8	**3**	614.7

^a^Catalytic data were obtained under similar conditions from three papers from Sasol researchers: **1**, **2**, **4, 5**, **30**, **32** from ref.^46^; **3** from ref.^44^; and **9**, **25**–**29**, and **31** from ref.^51^: 300 mL Parr reactor using 100 mL methylcyclohexane at 60 ˚C, 45–50 barg ethylene, 2.5–10 μmol Cr(acac)_3_, 1–1.5 eq. ligand, 270–480 eq. MMAO-3A.

As a qualitative summary of Fig. 7, the best-performing (meaning, in this case, the least polyethylene-producing) PNP ligands are those with ΔG_PPN_ > 6 kcal/mol, and we are heeding that in design and development of novel PNP-based catalysts. While unstable PNP ligands have been pre-coordinated to Cr to resist PPN isomerization^46^, we have chosen to only pursue the ligands with ΔG_PPN_ > 1 kcal/mol. Candidates **34**–**44**, ruled out by this criterion, are shown in Fig. 6, and their ΔG_PPN_ values are tabulated in Table 1. Significant researcher time will be saved by ruling out these candidates with non-trivial synthetic routes and poor expected catalytic performance.

Machine learning from quantum chemical computational models is a powerful tool that has often been brought to bear on ethylene oligomerization catalysts^52–56^. The scriptable and automated nature of our composite computational procedure is poised to contribute to catalyst discovery driven by machine learning and artificial intelligence. Unlike conventionally used molecular mechanics-based conformational searching algorithms, the extended tight binding theory used in our procedure is parameterized out-of-the-box for transition metals; an area of ongoing research is the application of XTBDFT toward the analysis of conformationally complex organometallic intermediates and transition states, perhaps using more computationally expensive DFT or coupled cluster methods.

## Conclusions

We have developed XTBDFT, an automated workflow to efficiently screen and evaluate conformationally complex molecules. We have applied this composite method to known PNP/PPN compounds to determine their relative thermodynamic stability and shown excellent agreement with the experimentally observed isomers. Furthermore, we show that thermodynamic stability of PNP ligands against isomerization to PPN is strongly correlated with lower undesired polyethylene formation. This procedure can be applied generally to other conformationally complex systems. This research leverages entirely open-source software, which we envision can be utilized by the greater computational chemistry and machine learning communities.

## Supplementary information

Supplementary nformation 1.

Supplementary information 2.

## Data Availability

Electronic Supplementary Information available: DFT-optimized Cartesian coordinates of molecules (.xyz) and table of calculated energies (.xlsx). Correspondence and other requests should be directed to S.L.
